# Workflow Efficiency in Vaginal Cuff High Dose Rate Brachytherapy Using Artificial Intelligence-Based Organ Segmentation and Multi-Channel Cylinder Modeling

**DOI:** 10.3390/cancers17172751

**Published:** 2025-08-23

**Authors:** Yohan A. Walter, Lane Rosen, Olivia Moncrief, Bethany Broekhoven, Troy Jacobs, Joseph Syh, Joseph Dugas, Kelsi Hoffnung, Mitchell Wolden, Heidi Wimberly, Jessica Nash, Melissa Camden, Daniel Speir, Krystal Jeffery, Philip Finley Durham, Kaylee Kallam, Hsinshun Terry Wu

**Affiliations:** 1 Department of Radiation Oncology, Willis Knighton Cancer Center, Shreveport, LA 71103, USAtwu@wkhs.com (H.T.W.); 2 Department of Clinical Research, University of Jamestown, Jamestown, ND 58405, USA; 3 School of Medicine, Louisiana State University Health Shreveport, Shreveport, LA 71103, USA

**Keywords:** brachytherapy, high dose rate, artificial intelligence, workflow efficiency, quality improvement, radiation oncology, vaginal cuff brachytherapy, treatment planning, radiation therapy

## Abstract

**Background/Objectives**: High dose rate brachytherapy (HDR-BT) is resource-intensive. Workflow efficiency and inter-user variability remain prevalent issues in HDR-BT. To improve workflow efficiency and reduce inter-user variability, we introduced artificial intelligence (AI)-based organ contouring (AC) and applicator modeling (AM) into our clinical workflow. Here, we present results on the impact of these tools on workflow efficiency, inter-user variability, and plan quality for vaginal cuff HDR-BT cases. **Methods**: 260 treated fractions were included in the analysis, half of which were treated before implementing AC and AM. Five different medical physicists performed the treatment planning. Using built-in timestamps, contouring, dose planning, and total treatment planning times were recorded for each fraction. Dosimetric data, including the dose to the highest 2cc (D2cc) of bladder and rectum, and percentage volumes of the target covered by 90–200% isodose lines (V90-200) were recorded. Analysis of variance with post-hoc Tukey tests were used to determine statistical differences between treatment planners before and after implementing AC and AM. **Results**: Implementing AC and AM resulted in an overall 13.7 ± 1.7-min reduction in planning time. Inter-user variability in organ and target dose metrics was reduced for most structures. Most notably, statistically significant differences in rectum D2cc and target V95 were observed between planners before, but not after, AC and AM implementation. **Conclusions**: AC and AM significantly improve workflow efficiency while maintaining plan quality. Reductions in inter-user variability and standardization of workflows may facilitate user training and robust outcome assessment.

## Simple Summary

High Dose Rate Brachytherapy is a key component of modern radiation oncology practice. However, brachytherapy is resource-intensive, requiring significant equipment, staffing, training, and time investment. Workflow efficiency and inter-user variability also impact current clinical practice and treatment plan quality. Our facility recently introduced two assistive planning tools which promise to improve workflow efficiency: artificial intelligence-assisted contouring and applicator modeling. Here, we present the results of 260 vaginal cuff high dose rate brachytherapy cases treated before and after implementing these planning tools to observe the impacts on workflow efficiency and treatment plan quality.

## 1. Introduction

Tumor control and survival benefits have been demonstrated for treatment protocols using high dose rate (HDR) brachytherapy (BT), compared to regimens using only external beam radiation therapy (EBRT), for management of several gynecological indications [1,2,3,4]. In the context of endometrial cancer, the PORTEC-2 trial demonstrated non-inferiority of vaginal BT compared to EBRT in overall survival or disease-free survival, though BT was associated with significantly lower rates of acute grade 1–2 gastrointestinal toxicity [5]. Similar results from other studies have solidified HDR-BT’s status as a treatment technique of choice for several gynecological indications [6,7,8,9,10,11,12,13,14].

Despite notable clinical benefits, the use of HDR-BT experienced a sharp decline in the 2000s among radiation oncology facilities in the United States [1,15,16]. A key factor leading to reductions in HDR-BT use was the advancement of EBRT [1,15]. EBRT presents a non-invasive alternative to HDR-BT, while advanced technologies like intensity modulation, and later particle beams, allow remarkable dose conformality in treatment planning. Additionally, a 2017 cost analysis performed by Bauer-Nilsen et al. [17] showed that EBRT required a four-fold lower attending physician time per relative value unit (RVU) compared to BT in treatment of cervical cancer, demonstrating the stronger relative cost effectiveness of EBRT for providers. The study pointed toward the coupling of heavy personnel requirements and the time-intensive nature of HDR-BT as key factors impacting end-to-end cost effectiveness [17].

Several attempts have been made to streamline the HDR-BT process. A 2014 study investigated the dosimetric impact and cost effectiveness of eliminating treatment replanning at each fraction [18]. The study revealed no significant dosimetric advantage to replanning, although there was approximately 20% greater cost effectiveness when using a common radioactive decay-corrected treatment plan for each fraction [18]. These findings were not surprising as treatment using decay-corrected plans sacrifices plan adaptation in exchange for greater efficiency [18]. However, effective workflows using common decay-corrected treatment plans should still feature verification processes before treatment to mitigate potential failure modes in applicator selection and placement, and to identify situations in which replanning may be necessary [19].

Several vendors have developed time-saving tools focused on improving treatment planning efficiency. These tools include applicator modeling (AM), in which the BT applicator is reconstructed based on a pre-defined template provided by the vendor, and artificial intelligence (AI)-assisted organ segmentation, in which organs at risk (OARs) are automatically contoured based on provided images. As organ segmentation and applicator reconstruction are both crucial, yet time-consuming steps in the BT workflow, both tools may significantly improve treatment planning efficiency, while also improving standardization and accuracy of organ and applicator delineation.

At the Willis Knighton Cancer Center (Shreveport, Louisiana, USA), our gynecological HDR-BT program follows a streamlined workflow involving computed tomography (CT)-based daily replanning. In 2023, we introduced both AM and AI-based autocontouring (AC) into our clinical workflow. The purpose of this study is to assess the impact of these automated tools on treatment planning efficiency, inter-user variability, and plan dosimetry. The results herein represent real-world data from a community cancer center, which may be used to inform future brachytherapy practice in radiation oncology clinics.

## 2. Materials and Methods

### 2.1. Patient Cohort

Patients receiving vaginal cuff HDR-BT between 2022 and 2025 using a multi-channel vaginal cylinder (MCVC) were included. Prescription dose regimens were 1800 cGy in 4 fractions, 2200 cGy in 4 fractions, 1500 cGy in 3 fractions, or 2100 cGy in 3 fractions. All treatments were prescribed to a 5.0 mm depth with a 40 or 50 mm active length. Patients were treated with HDR-BT alone or in conjunction with photon or proton-based EBRT. Non-vaginal cuff HDR-BT cases were excluded from this analysis.

### 2.2. Applicator Placement

All vaginal cuff HDR-BT treatments were delivered on an outpatient basis. Prior to patient arrival, a medical physicist assembled the MCVC (Vaginal CT/MR Multi Channel Applicator; Elekta AB, Stockholm, Sweden) and recorded applicator channel lengths for treatment planning. Upon arrival, patients were brought to the procedure room, which houses examination equipment, positioning devices, applicators, patient transport systems, and a mobile CT unit (BodyTom; Samsung-NeuroLogica, Danvers, MA, USA) dedicated to HDR-BT procedures. Before applicator placement, the physician assistant performed a gynecological examination. If the results of the clinical assessment were acceptable, the physician or physician assistant placed the applicator. The largest tolerable cylinder was used for optimal plan dosimetry. Available cylinder diameters were 2.5, 3.0, and 3.5 cm.

After MCVC placement, the position of the applicator was marked on the patient’s skin. A CT scan was then acquired for positioning verification and as the basis for treatment planning. The physician, physician assistant, CT operator (technologist or radiation therapist), and medical physicist verified the applicator placement and acceptable image quality. The CT image set was exported to the BT treatment planning system (Oncentra Brachy, version 4.6.0 to 4.6.3; Elekta AB, Stockholm, Sweden).

### 2.3. Treatment Planning Before AC and AM

**Figure 1:**
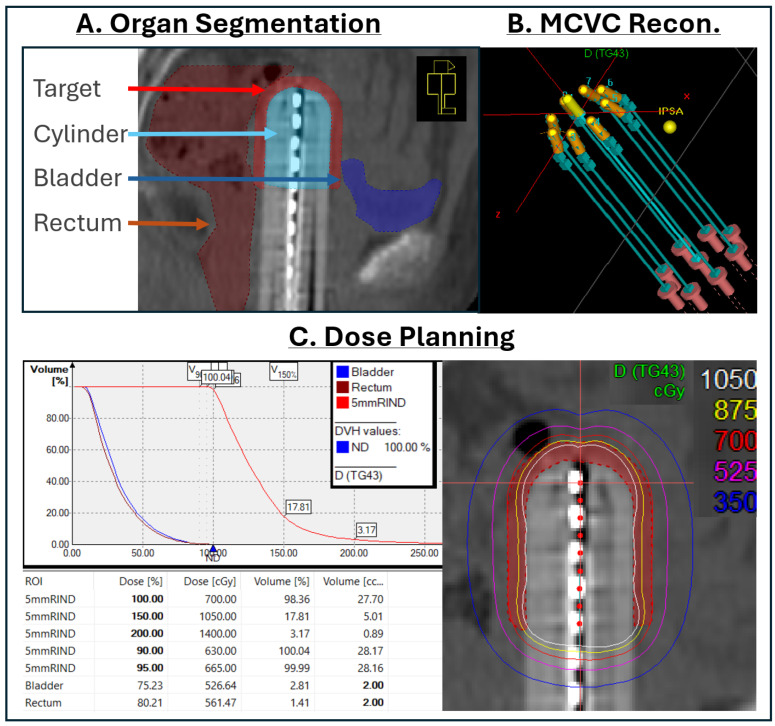
Vaginal cuff brachytherapy treatment planning workflow without AI autocontouring and applicator modeling. Step (**A**): CT images are imported. Bladder, rectum, and target (5 mm rind surrounding the cylinder) are contoured. Step (**B**): The applicator is reconstructed manually. Channel numbers are indexed and measured lengths are entered. Step (**C**): Dwell positions are activated using inverse planning simulated annealing (IPSA) and the dose distribution is further modified to meet objectives before plan approval.

Treatment planning was performed by five medical physicists. The planning workflow is detailed in Figure 1.

Following applicator placement, the CT scan was imported into the planning system, and the bladder, rectum, and target were contoured. The target volume was a uniform 5.0 mm-thick rind surrounding the MCVC, extending from 5.0 mm beyond the end of the cylinder to the end of the specified active length (4.0 or 5.0 cm).

After OAR and target delineation, applicator reconstruction was performed. The 2.5 cm-diameter MCVC had six peripheral and a central channel, while the 3.0 and 3.5 cm MCVCs each had eight peripheral and one central channel. The orientation of the applicator was verified prior to reconstruction of individual channels. The channel length, position offset, and index numbers were entered into the planning system.

Inverse planning simulated annealing (IPSA) was used for initial dwell position activation and dose optimization. The IPSA objectives featured a pre-defined prescription dose, target coverage, and OAR dose goals. Dwell positions for all channels were activated in 5.0 mm increments from the end of each channel to the end of the active treatment length. Users then manually adjusted dose distributions such that planning objectives were met. The highest dose to 2 cc (D2cc) of the bladder and rectum were kept within 80% of the prescription dose, though higher doses were allowed in some cases for which EBRT was not used, at the discretion of the physician. Similarly, at least 95% of the target volume was covered by the 95% isodose line (V95). In optimization, planners prioritized maximum target coverage while remaining within OAR constraints.

The physician then reviewed the treatment plan, and if required, adjusted the dose distribution before plan approval. After review, the approved plan was sent to the treatment delivery system (Flexitron; Elekta AB, Stockholm, Sweden) and another physicist performed a systematic double-check of the treatment plan. Following secondary physicist review, the treatment was delivered by a radiation therapist and physicist, the physician assistant removed the applicator, and the patient was discharged from the facility.

### 2.4. Treatment Planning After Implementing AC and AM

**Figure 2:**
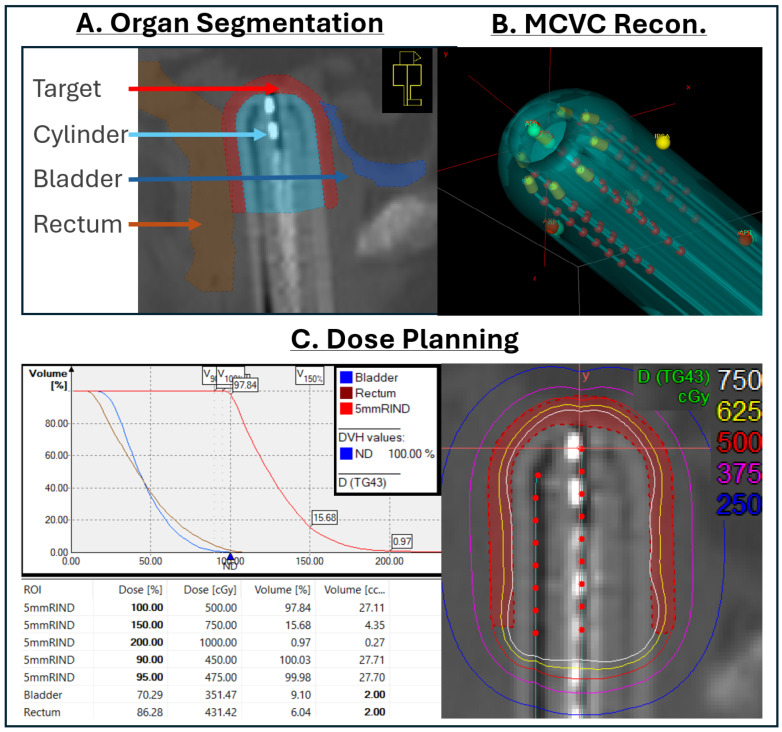
Vaginal cuff brachytherapy treatment planning workflow using AI autocontouring and applicator modeling. Step (**A**): CT images with AI-generated bladder and rectum structures are imported. The target (5 mm rind surrounding the cylinder) is manually contoured. Bladder and rectum contours are reviewed for accuracy and modified as needed. Step (**B**): The applicator is reconstructed using applicator modeling. Model placement is verified and adjusted as needed. Channel numbers are indexed and measured lengths are entered. Step (**C**): Dwell positions are activated using inverse planning simulated annealing (IPSA) and the dose distribution is further modified to meet objectives before plan approval.

The treatment planning workflow after implementing AC and AM is summarized in Figure 2.

Following applicator placement, the CT image set was processed in Limbus Contour Version 1.8 (Limbus AI; Radformation, New York, NY, USA) for AI-based autosegmentation. Contours for the bladder and rectum were generated using the gynecological brachytherapy-specific anatomical models and imported into the planning system. The contours were reviewed by the physicist and edited if necessary. Target volumes were contoured manually.

Applicator reconstruction was performed using library models within the planning system. The physicist selected the applicator model corresponding with the MCVC being used. The model was positioned by placing at least four “anchor” points in specified positions on the applicator in the CT image. The final placement was verified by the physicist. Finally, the channel lengths and index numbers were entered before proceeding to the dose optimization, plan approval, and treatment delivery steps, which mirrored the processes outlined in Section 2.3.

### 2.5. Data Collection

Five medical physicists each generated at least ten vaginal cuff HDR-BT treatment plans before and after implementing both AC and AM. The MCVC diameter, prescription dose, and active treatment length were recorded for each plan.

Efficiency data were collected by recording timestamps correlating with each step of the treatment planning workflow. The timestamps included CT scan acquisition, completion of OAR and target contours, and plan approval. The time between CT scan acquisition and contour completion (contouring time), time between contour completion and plan approval (dose planning time), and time between CT scanning and plan approval (total planning time) were calculated for each treated fraction.

Dosimetric data were collected to assess the influence of automated planning steps on inter-user variability and plan quality. The D2cc for the bladder and rectum, and the percentage of the target volume receiving at least 90%, 95%, 100%, 150%, and 200% of the prescription dose (V90, V95, V100, V150, V200, respectively), were recorded.

To reduce the influence of planners acclimating to the assistive planning tools, efficiency and dosimetric data for the first five treatment plans generated after implementing AC and AM were discarded for each physicist. Additionally, treatment plans generated while mentoring medical physics residents were not included in the analysis.

### 2.6. Data Analysis

Descriptive statistics were performed to assess central tendency and dispersion. One-way analysis of variance (ANOVA) was used to assess statistical differences in planning efficiency between physicists, dosimetric parameters between physicists, and dosimetric parameters between selected applicators. The F-statistic, *p*-value, and ANOVA effect size, ω^2^, were reported. The value of ω^2^ corresponds with the proportion of variance in the dependent variable accounted for by the independent variable, taking values between ±1 [19]. A value of 0 indicates no effect, while nonzero effect sizes were interpreted as large (≥0.15), moderate (0.06–0.14), or small (≤0.05) [20]. Post-hoc Tukey Honestly Significant Difference (Tukey HSD) tests were performed for pairwise comparisons.

Independent samples two-sided t-tests were used to compare dosimetric and planning time metrics between treatment plans generated before and after implementing AC and AM, and to assess the influence of active treatment length on plan dosimetry. Statistical significance was taken as *p* < 0.05. All data analysis was performed in STATA version 18.0 (StataCorp., College Station, TX, USA).

## 3. Results

Treatment planning data for 93 patients undergoing vaginal cuff HDR-BT were collected. 260 total treatment plans were analyzed. 130 of the included plans (50 patients) were generated before introducing AC and AM. 130 plans (43 patients) generated after implementation were included in the analysis. The distribution of prescription doses, applicator choices, and planners are listed in Table 1.

### 3.1. Treatment Planning Efficiency

The overall average treatment planning time across all 260 fractions was 46.6 ± 15.2 min. Contouring and dose planning processes had nearly equal contributions to the total planning time, averaging 23.5 ± 10.0 and 23.6 ± 8.9 min, respectively. Table 2 summarizes the influence of AC and AM tools on planning time for five medical physicists.

Significant differences in contouring time were observed between planners before (F(4,125) = 11.35, *p* < 0.01, ω^2^ = 0.24) and after introducing AC (F(4,125) = 28.25, *p* < 0.01, ω^2^ = 0.46). Implementing AC most dramatically reduced contouring time for planner 2, who had an average 7.4 ± 2.3-min reduction in time between CT scan acquisition and contour completion (*p* = 0.05, 95% CI: 0.0 to 14.9 min). Across all planners, the time from scan to contour completion was reduced by 6.8 ± 1.2 min (*p* < 0.01, 95% CI: 4.5 to 9.1 min).

Statistical differences in dose planning time were observed between planners before (F(4,125) = 5.48, *p* < 0.01, ω^2^ = 0.12) and after introducing AM (F(4,125) = 14.61, *p* < 0.01, ω^2^ = 0.30). Planner 5 had the most dramatic decrease in dose planning time with implementation of AM, which reduced planning time by 9.3 ± 1.6 min (*p* < 0.01, 95% CI: 1.0 to 14.4 min). Across all planners, the dose planning time was reduced by 7.0 ± 1.0 min (*p* < 0.01, 95% CI: 5.0 to 9.0 min).

Large and statistical differences in total planning time were observed between planners before (F(4,125) = 16.06, *p* < 0.01, ω^2^ = 0.32) and after introducing both AM and AC (F(4,125) = 36.21, *p* < 0.01, ω^2^ = 0.52). Post-hoc analysis showed that, prior to implementing AC and AM, the biggest gap in total planning time was 25.3 ± 3.5 min (*p* < 0.01, 95% CI: 14.1 to 36.4 min), between physicists 1 and 3. After introducing AC and AM, the biggest difference was observed between planners 1 and 5, at 27.6 ± 3.1 min (*p* < 0.01, 95% CI: 17.7 to 37.5 min). The difference in planning time between physicists 1 and 3 slightly increased when using AC and AM, at 26.3 ± 3.2 min (*p* < 0.01, 95% CI: 15.9 to 36.6 min). Differences were not statistically significant between planners 3, 4, and 5 before or after implementation (*p* > 0.40 for all pairwise comparisons).

Averaged over all fractions, the total planning time decreased by 13.7 ± 1.7 min (*p* < 0.01, 95% CI: 11.3 to 17.0 min). Planners 2 and 5 had the most significant overall time savings, reducing mean total planning time by 14.7 ± 3.1 min (*p* < 0.01, 95% CI: 4.9 to 24.6 min) and 16.2 ± 2.4 min (*p* < 0.01, 95% CI: 8.8 to 23.6 min), respectively. Though each physicist experienced at least marginal reductions in planning time, planner 1 had the smallest efficiency gain, cutting 7.3 ± 3.7 min from their average time. Applicator modeling led to a nearly negligible reduction in dose planning time for planner 1.

### 3.2. Organ Dose

Statistically significant differences in bladder D2cc were observed between planners before (F(4,125) = 6.57, *p* < 0.01, ω^2^ = 0.15) and after implementing AC and AM (F(4,125) = 3.71, *p* < 0.01, ω^2^ = 0.08). The average D2cc was 13.1 ± 3.5% lower for planner 5 compared to planner 2 before AC and AM (*p* < 0.01, 95% CI 4.2 to 22.9%). After implementation, the difference reduced to 11.7 ± 3.3% (*p* = 0.02, 95% CI: 1.2 to 22.2%).

On average, the D2cc for bladder reduced by 3.1 ± 1.6% with AC and AM, though the difference was not statistically significant (*p* = 0.05). None of the planners had a statistically significant difference in bladder D2cc with AC and AM, though all showed slight decreases in average D2cc (Table 3).

The bladder D2cc was significantly affected by the applicator size (F(2,257) = 4.99, *p* < 0.01, ω^2^ = 0.03) and the prescribed active length (*p* < 0.01). Plans using a 3.5 cm diameter MCVC were associated with 4.9 ± 1.7% and 5.5 ± 2.2% higher bladder D2cc as compared to 3.0 (*p* = 0.01, 95% CI: 0.8 to 9.0%) and 2.5 cm (*p* = 0.04, 95% CI: 0.3 to 10.8%) diameter cylinders, respectively (Table A1). A 5.0 cm active length increased average bladder D2cc by 4.7 ± 1.7% (*p* < 0.01, 95% CI: 1.3 to 8.0%, Table A2).

Planning with AC and AM reduced rectum D2cc by 6.0 ± 1.2%, averaged over all cases. Statistically significant differences in rectum D2cc were observed between planners before (F(4,125) = 7.47, *p* < 0.01, ω^2^ = 0.17), but not after implementing AC and AM (F(4,125) = 1.43, *p* = 0.23, ω^2^ = 0.01). The biggest differences in rectum D2cc were observed between planners 1 and 3 before AC and AM (*p* < 0.01, 95% CI: 1.6 to 21.7%), and between planners 1 and 2 after AC and AM (*p* = 0.61, 95%CI: −3.0 to 14.8%).

Dramatic reductions in rectum D2cc were observed for planners 2, 4, and 5 (Table 3), though the differences were statistically significant for planners 4 (*p* < 0.01, 95%CI: 1.5 to 18.9%) and 5 (*p* < 0.01, 95% CI: 2.4 to 15.6%). The rectum D2cc was significantly affected by the applicator diameter (F(2,257) = 7.09, *p* < 0.01, ω^2^ = 0.04) and active treatment length (*p* < 0.01). Use of the 3.5 cm diameter MCVC increased D2cc by 5.5 ± 1.8 and 4.5 ± 1.4% relative to the 2.5 (*p* < 0.01, 95%CI: 1.3 to 9.7%) and 3.0 cm (*p* < 0.01, 95% CI: 1.3 to 7.8%) cylinders, respectively (Table A1). A 5.0 cm active treatment length increased the rectum D2cc by 5.4 ± 1.4% as compared to a 4.0 cm active length (*p* < 0.01, 95% CI: 2.7 to 8.0%, Table A2).

### 3.3. Target Dose

The effects of implementing AC and AM on target V90, V95, V100, V150, and V200 are summarized in Table 4 and Table A3.

Averaged over all planners, implementing AC and AM had a nearly negligible impact on V95 (*p* = 0.95). The overall mean V95 was 98.6 ± 3.7%. Small, but statistically significant differences in V95 were observed between planners before (F(4,125) = 2.79, *p* = 0.03, ω^2^ = 0.05), but not after using AC and AM (F(4,125) = 1.43, *p* = 0.23, ω^2^ = 0.01). V95 was not significantly influenced by the choice of applicator (F(2,257) = 1.32, *p* = 0.27, ω^2^ = 0.002). A 4.0 cm active treatment length was associated with a 1.3 ± 0.5% higher V95 as compared to a 5.0 cm length (*p* < 0.01, Table A2).

Introducing AC and AM did not significantly affect V100 (*p* = 0.81, Table 4), averaged over all planners. Additionally, there were no statistically significant differences in V100 between planners before (F(4,125) = 0.66, *p* = 0.62, ω^2^ = −0.01) or after (F(4,125) = 1.74, *p* = 0.15, ω^2^ = 0.02) implementing AC and AM. No statistically significant dependence of V100 on applicator size was observed (F(2,257) = 0.85, *p* = 0.43, ω^2^ = −0.001, Table A1). Plans using a 4.0 cm active length averaged 3.0 ± 1.0% higher V100 compared to the 5.0 cm active length (Table A2).

An overall decrease in V150 was observed when using AC and AM (*p* < 0.01, Table 4). The most dramatic change was observed for planner 1, where average V150 dropped by 5.8 ± 2.0%, though the difference was not statistically significant (*p* = 0.11). There was a unanimous, but not statistically significant drop in average V150 for each planner after introducing AC and AM. No significant differences in V150 were observed after AC and AM introduction. V150 and V200 were both significantly affected by the applicator size (F(2,257) = 7.89, *p* < 0.01, ω^2^ = 0.05; F(2,257) = 10.43, *p* < 0.01, ω^2^ = 0.07, respectively). In both cases, the difference between 2.5 and 3.0 cm cylinders was not statistically significant, while the 3.5 cm cylinder was associated with lower target V150 and V200 (*p* < 0.01, Table A1). The active treatment length did not significantly impact V150 (*p* = 0.57, Table A2).

Statistically significant, though slight differences were observed V200 following AC and AM implementation (*p* < 0.01, Table A3). Differences in V90 were not statistically significant (*p* = 0.84). V90 and V200 averaged 99.4 ± 2.3% and 1.8 ± 1.3%, respectively, across all fractions. The 5.0 cm active treatment length was associated with a slight, but statistically significant 0.8 ± 0.3% drop in V90 (*p* < 0.01). V200 was not significantly affected by the active length (*p* = 0.45).

## 4. Discussion

The clinical and dosimetric benefits of gynecological HDR-BT procedures have solidified its role as an effective and crucial modality in a comprehensive radiation oncology clinic. Effective vaginal cuff HDR-BT procedures are time-sensitive, yet time-intensive, and require specially trained clinical and technical staff [19,21,22,23]. The purpose of this study was to quantify the workflow efficiency gains in using AC and AM, and to assess the influence of these tools on treatment plan quality and inter-user variability. To investigate the impact of these assistive planning tools, we collected treatment data from 260 patients treated with vaginal cuff HDR-BT performed in our clinic. To our knowledge, this is the first study to report workflow efficiency and dosimetric impacts of AI-assisted autosegmentation and BT applicator modeling across multiple treatment planners.

Our results demonstrated significant efficiency gains with use of AC and AM in our treatment planning workflow (Table 2). The implementation of AC and AM led to similar improvements in planning efficiency in the respective workflow steps (contouring and dose planning, respectively), resulting in a significant 13.7 ± 1.7-min savings in planning time. Unanimous, though not always statistically significant, efficiency gains were demonstrated for all five treatment planners, demonstrating durability of the effect across different subjects. When averaged across all treated fractions, the efficiency gains in each workflow step were statistically significant (*p* < 0.01).

Dosimetric improvements were visualized in the D2cc for both the bladder and rectum. Though the bladder D2cc was not statistically different before and after AC and AM implementation, the average D2cc decreased for both OARs. These results may represent a coupled effect between changes in organ segmentation and possible increased user input in manipulation of dose distributions. The reduction in organ dose while retaining consistent average V95 and V100 may indicate improvements in dosimetric plan quality, though correlation with clinical outcomes may be merited in future studies.

Statistical differences were observed in rectum D2cc, target V95, and V150 between planners before introducing AC and AM. Differences in each metric were not statistically significant after implementation, signaling a reduction in inter-user variability in these metrics. The target V100 was consistent between planners before and after using AC and AM, signaling unanimous prioritization of target coverage. There was also improved agreement in V95 between planners which may signal reduced variability in organ contouring, thus generating more consistent tradeoffs between target coverage and OAR dose in treatment plan optimization. However, an influence on the effect may have been the increased dispersion in V95 and V100 for planners 1 and 4 when using AC and AM (Table 4), which partly stemmed from three cases (nine fractions) in which meeting OAR dose constraints was prioritized more heavily than maximizing target coverage. Statistical differences in bladder D2cc were observed between planners before and after introducing AC and AM, though the effect size was smaller after implementation, signaling potential improvements in inter-user variability despite visualized differences between planners.

The time-intensive nature of HDR-BT for the vaginal cuff stems from multiple factors, including staffing stress, patient comfort, and physiological processes [19,21,22,23,24]. The typical image-guided BT workflow involves organ segmentation and dose planning based on pre-treatment magnetic resonance (MRI) or CT images [19]. Since the vaginal cuff is adjacent to the bladder and rectum, anatomical structures known to change in shape over time due to filling [25], representative organ dosimetry may only be achievable by minimizing the gap between image acquisition and treatment delivery [25,26]. The nearly 15-min average gain in workflow efficiency may result in more consistent agreement between anatomy during pre-planning scans and in treatment delivery, though future studies are needed to characterize anatomical changes over time in the vaginal cuff HDR-BT context. Additionally, workflow efficiency improvements were achieved via introduction of AC and AM, while still delivering comparable plan quality, indicating viability of the assisted treatment planning workflow in a busy clinical environment.

Inter-user variability remains a challenge across the field, especially for facilities in which multiple staff handle clinical cases on a rotating basis [27,28]. Standardization of procedures ensures consistency between rotating staff, while potentially reducing impact of potential failure modes in clinical workflows [19,21,29,30]. Our findings suggest that automated and assistive tools, like AC and AM, have the potential to improve consistency between observers and facilitate the development of stronger standard operating procedures (SOPs). Enhancing the quality of SOPs, paired with automation in treatment planning, may expedite comprehensive training of new staff, or cross-training of other staff, in HDR-BT treatment planning.

The advanced practice radiation therapist (APRT) has been described extensively in the literature [31,32,33,34] and typically involves designation and training of a radiation therapist as a specialist in a particular clinical area. Staff training has been identified as a major hurdle in developing advanced clinical roles for radiation therapists [31]. Established APRT roles include clinical practice leadership [31,32,33], technical support for online adaptive radiation therapy programs [32,35], and BT [34,36,37,38]. Reported APRT roles specific to BT primarily include pre-treatment patient examinations, applicator placement, organ segmentation, and overall clinical process administration [34,36,37]. Specific BT-specialized APRT responsibilities vary by facility and typically have not involved dose planning processes.

Tools like AI-assisted organ segmentation, applicator modeling, and library template-based dose planning may significantly accelerate clinical workflows, while expanding APRT involvement in critical aspects of the BT workflow by facilitating cross-training and standardizing workflows [31,39]. Additionally, standardized BT-specialized APRT roles may be developed, in which responsibilities and core competencies are more clearly defined, which may improve consistency in practice while enhancing patient safety. Development of BT-specialized APRT roles may be especially beneficial for facilities lacking sufficient on-site physics and dosimetry support, such as clinics in rural or low and middle-income areas.

A limitation of this work is that the influence of AC and AM on plan dosimetry and workflow efficiency could not be decoupled based on the presented data. For example, workflow efficiency improvements may drive planners to dedicate more time to plan optimization, resulting in smaller efficiency gains, but potential dosimetric improvements. Additionally, the use of AC may influence resultant contours, which impacts dosimetry results [40,41,42,43,44]. The contouring time also varied significantly between physicists, indicating that each planner may dedicate different amounts of time toward refining AI-generated contours. Furthermore, planners had varying levels of experience and case load in the analysis (Table 1), which may impact interpretation of results. Planners 4 and 5 had the least clinical experience (5 years and 3 years at study conclusion, respectively), and therefore, some workflow efficiency and plan quality metrics were likely influenced by growing expertise. Variability between patients treated before and after implementing AC and AM was also a potential confounding factor, as the comparisons were made across independent cases. However, the selected study design guarantees similar user input and plan quality, as all compared treatment plans were clinically utilized and were generated under the prevailing standard workflow. Finally, two different physicians reviewed treatment plans, potentially further impacting inter-user variability. Future work investigating individual patterns of use and correlating clinical outcomes with plan dosimetry before and after implementing automated tools may be necessitated to fully characterize the effects.

## 5. Conclusions

Workflow efficiency and standardization are critical in HDR-BT. Patient comfort, staffing, and dosimetry may all be affected by procedure time. Inter-user variability may also affect treatment plan quality, and therefore clinical outcome. It is thus paramount that efficient workflows be designed while reducing the influences of inter-user variability. Based on our clinical experience, AC and AM significantly improve treatment planning efficiency, while retaining excellent plan quality. Effects of inter-user variability were reduced, highlighting the value of assistive planning tools on standardization across treatment planners. These results may support the expansion of APRT roles in BT, and AC and AM may be considered as tools for improving workflow efficiency, which may be especially beneficial in high-volume tertiary referral BT clinics.

## Figures and Tables

**Table 1:** Summary of included cases and distribution of treatment planners before and after implementing AI-based autocontouring (AC) and applicator modeling (AM).

Variable	Subgroup	Number of Fractions	Percentage
**Total**		**260**	**100.0**
**Prescription Dose (cGy)**	450	7	2.7
	500	134	51.5
	550	22	8.5
	700	97	37.3
**Active Length (cm)**	4.0	183	70.4
	5.0	77	29.6
**MCVC Diameter (cm)**	2.5	47	18.1
	3.0	117	45.0
	3.5	96	36.9
**Post-AC, AM**	**Total**	**130**	**50.0**
	Planner 1	16	12.3
	Planner 2	20	15.4
	Planner 3	30	23.1
	Planner 4	25	19.2
	Planner 5	39	30.0
**Pre-AC,** **AM**	**Total**	**130**	**50.0**
	Planner 1	17	13.1
	Planner 2	27	20.8
	Planner 3	19	14.6
	Planner 4	23	17.7
	Planner 5	44	33.8

Abbreviation: MCVC, multi-channel vaginal cylinder.

**Table 2:** Contouring, dose planning, and total planning time before and after implementing AI-based autocontouring (AC) and applicator modeling (AM).

Planner No.	Contouring Time (SD) [min]			Dose Planning Time (SD) [min]			Total Planning Time (SD) [min]		
	Pre-AC/AM	Post-AC/AM	*p*	Pre-AC/AM	Post-AC/AM	*p*	Pre-AC/AM	Post-AC/AM	*p*
Planner 1	36.6 (13.7)	30.6 (9.5)	*0.46*	31.8 (3.8)	30.6 (6.9)	*1.00*	68.4 (12.9)	61.1 (10.9)	*0.60*
Planner 2	33.3 (12.6)	25.9 (5.1)	*0.05*	31.0 (8.4)	23.8 (10.0)	*0.03*	62.1 (12.9)	47.4 (8.2)	*<0.01*
Planner 3	19.9 (9.6)	16.3 (2.8)	*0.86*	23.3 (7.8)	18.6 (5.4)	*0.46*	43.2 (13.6)	34.8 (6.6)	*0.18*
Planner 4	22.6 (7.7)	17.5 (6.3)	*0.44*	25.2 (8.2)	18.2 (9.0)	*0.03*	47.8 (12.7)	35.7 (12.3)	*<0.01*
Planner 5	24.5 (6.4)	17.3 (4.2)	*<0.01*	25.5 (7.8)	16.2 (3.8)	*<0.01*	49.7 (9.7)	33.5 (6.7)	*<0.01*
All	26.9 (11.2)	20.1 (7.4)	*<0.01*	27.1 (8.1)	20.0 (8.2)	*<0.01*	53.4 (14.5)	39.8 (12.7)	*<0.01*

**Table 3:** The dose to the highest 2 cc (D2cc) of contoured bladder and rectum separated by planner before and after implementing AI-based autocontouring (AC) and applicator modeling (AM). Values are given as percentages of the prescription dose to match clinical treatment planning goals.

Planner No.	Bladder D2cc (SD) [%]			Rectum D2cc (SD) [%]		
	Pre-AC/AM	Post-AC/AM	*p*	Pre-AC/AM	Post-AC/AM	*p*
Planner 1	83.0 (18.2)	79.0 (9.3)	*0.99*	88.2 (9.5)	84.8 (14.8)	*0.99*
Planner 2	85.6 (7.4)	81.6 (5.0)	*0.98*	87.0 (9.7)	78.5 (9.6)	*0.08*
Planner 3	77.0 (6.5)	73.4 (11.5)	*0.99*	76.5 (8.8)	82.1 (6.7)	*0.59*
Planner 4	76.5 (12.8)	76.0 (18.0)	*1.00*	89.2 (9.2)	79.1 (10.9)	*<0.01*
Planner 5	72.1 (11.8)	69.9 (12.1)	*1.00*	87.9 (7.0)	78.9 (10.0)	*<0.01*
All	77.9 (12.7)	74.8 (12.8)	*0.05*	86.3 (9.4)	80.3 (10.3)	*<0.01*

**Table 4:** Target coverage dose separated by planner before and after implementing AI-based autocontouring (AC) and applicator modeling (AM). Values are given as the percentage volume of the target receiving the specified dose level relative to the prescription.

Planner No.	V95 (SD) [%]			V100 (SD) [%]			V150 (SD) [%]		
	Pre-AC/AM	Post-AC/AM	*p*	Pre-AC/AM	Post-AC/AM	*p*	Pre-AC/AM	Post-AC/AM	*p*
Planner 1	97.3 (4.1)	97.3 (6.8)	*1.00*	94.9 (6.9)	93.9 (8.3)	*1.00*	20.5 (9.5)	14.7 (3.5)	*0.11*
Planner 2	99.3 (1.8)	99.4 (1.2)	*1.00*	97.1 (3.5)	96.9 (2.4)	*1.00*	15.7 (3.1)	15.7 (2.7)	*1.00*
Planner 3	97.1 (4.5)	98.6 (1.1)	*0.88*	93.2 (7.2)	95.5 (2.5)	*0.99*	19.3 (11.8)	18.1 (4.0)	*0.99*
Planner 4	99.7 (0.7)	97.8 (5.7)	*0.66*	94.8 (18.7)	94.9 (7.3)	*1.00*	22.5 (8.1)	19.0 (6.8)	*0.54*
Planner 5	98.9 (3.7)	99.3 (1.7)	*1.00*	96.5 (5.1)	97.0 (3.5)	*1.00*	17.5 (3.4)	15.9 (2.8)	*0.96*
All	98.6 (3.3)	98.6 (3.7)	*0.95*	95.6 (9.3)	95.9 (5.0)	*0.81*	18.7 (7.3)	16.8 (4.4)	*0.01*

## Data Availability

The data supporting the findings of this study are available from the corresponding author upon reasonable request.

## Abbreviations

The following abbreviations are used in this manuscript:

HDR	High dose rate
BT	Brachytherapy
EBRT	External beam radiation therapy
RVU	Relative value unit
AM	Applicator modeling
AI	Artificial intelligence
OAR	Organ-at-risk
CT	Computed Tomography
AC	AI-based autocontouring
MCVC	Multi-channel vaginal cylinder
IPSA	Inverse planning simulated annealing
DXcc	Dose to the highest “X”cc of a segmented structure as percentage of prescribed dose
VXX	Percentage volume of a segmented structure covered by the “XX” percentage isodose line
ANOVA	Analysis of variance
Tukey HSD	Tukey’s Honestly Significant Difference test
MRI	Magnetic resonance imaging
APRT	Advanced practice radiation therapist

## Appendix A

**Table A1:** D2cc of bladder and rectum and target coverage metrics sorted by multi-channel vaginal cylinder (MCVC) applicator size.

Structure	2.5 cm MCVC	3.0 cm MCVC	3.5 cm MCVC	*p*
Bladder D2cc (SD) [%]	74.0 (13.5)	74.6 (13.9)	79.5 (10.2)	*<0.01*
Rectum D2cc (SD) [%]	80.8 (8.8)	81.8 (11.0)	86.3 (9.4)	*<0.01*
Target V90 (SD) [%]	98.9 (3.7)	99.4 (2.3)	99.7 (1.0)	*0.18*
Target V95 (SD) [%]	98.0 (5.4)	98.6 (3.5)	99.0 (2.0)	*0.27*
Target V100 (SD) [%]	95.5 (7.2)	95.2 (9.5)	96.5 (3.7)	*0.43*
Target V150 (SD) [%]	19.2 (5.7)	18.7 (6.1)	15.8 (5.7)	*<0.01*
Target V200 (SD) [%]	2.2 (1.4)	2.1 (1.4)	1.4 (1.0)	*<0.01*

**Table A2:** D2cc of bladder and rectum and target coverage metrics sorted by active treatment length.

Structure	4.0 cm Active Length	5.0 cm Active Length	*p*
Bladder D2cc (SD) [%]	74.9 (13.1)	79.6 (11.5)	*<0.01*
Rectum D2cc (SD) [%]	81.7 (8.8)	87.1 (12.4)	*<0.01*
Target V90 (SD) [%]	99.7 (1.0)	98.8 (3.9)	*<0.01*
Target V95 (SD) [%]	99.0 (2.1)	97.7 (5.5)	*<0.01*
Target V100 (SD) [%]	96.7 (3.8)	93.6 (12.1)	*<0.01*
Target V150 (SD) [%]	17.9 (4.5)	17.4 (8.7)	*0.57*
Target V200 (SD) [%]	1.8 (1.2)	1.9 (1.6)	*0.45*

**Table A3:** Additional target coverage dose separated by planner before and after implementing AI-based autocontouring (AC) and applicator modeling (AM). Values are given as the percentage volume of the target receiving the specified dose level relative to the prescription.

Planner No.	V90 (SD) [%]			V200 (SD) [%]		
	Pre-AC/AM	Post-AC/AM	*p*	Pre-AC/AM	Post-AC/AM	*p*
Planner 1	99.1 (1.7)	98.4 (5.6)	*1.00*	2.7 (2.4)	1.2 (0.5)	*0.02*
Planner 2	99.8 (0.8)	99.9 (0.4)	*1.00*	1.6 (0.9)	1.5 (0.8)	*1.00*
Planner 3	98.7 (2.5)	99.5 (0.5)	*0.96*	1.2 (1.1)	1.7 (1.0)	*0.95*
Planner 4	99.9 (0.3)	98.9 (3.8)	*0.84*	2.7 (1.4)	1.5 (1.6)	*0.03*
Planner 5	99.4 (2.6)	99.8 (0.6)	*1.00*	2.3 (1.4)	1.7 (0.9)	*0.46*
All	99.4 (1.9)	99.4 (2.6)	*0.84*	2.1 (1.5)	1.6 (1.0)	*<0.01*
